# The Role of ^18^F-FDG PET/CT Integrated Imaging in Distinguishing Malignant from Benign Pleural Effusion

**DOI:** 10.1371/journal.pone.0161764

**Published:** 2016-08-25

**Authors:** Yajuan Sun, Hongjuan Yu, Jingquan Ma, Peiou Lu

**Affiliations:** 1 Department of Radiological Diagnosis, The Affiliated Tumor Hospital of Harbin Medical University, Harbin, Heilongjiang Province, China; 2 Department of hematology, The First Affiliated Hospital of Harbin Medical University, Harbin, Heilongjiang Province, China; 3 Center of PET/CT, The Affiliated Tumor Hospital of Harbin Medical University, Harbin, Heilongjiang Province, China; Wayne State University, UNITED STATES

## Abstract

**Objective:**

The aim of our study was to evaluate the role of ^18^F-FDG PET/CT integrated imaging in differentiating malignant from benign pleural effusion.

**Methods:**

A total of 176 patients with pleural effusion who underwent ^18^F-FDG PET/CT examination to differentiate malignancy from benignancy were retrospectively researched. The images of CT imaging, ^18^F-FDG PET imaging and ^18^F-FDG PET/CT integrated imaging were visually analyzed. The suspected malignant effusion was characterized by the presence of nodular or irregular pleural thickening on CT imaging. Whereas on PET imaging, pleural ^18^F-FDG uptake higher than mediastinal activity was interpreted as malignant effusion. Images of ^18^F-FDG PET/CT integrated imaging were interpreted by combining the morphologic feature of pleura on CT imaging with the degree and form of pleural ^18^F-FDG uptake on PET imaging.

**Results:**

One hundred and eight patients had malignant effusion, including 86 with pleural metastasis and 22 with pleural mesothelioma, whereas 68 patients had benign effusion. The sensitivities of CT imaging, ^18^F-FDG PET imaging and ^18^F-FDG PET/CT integrated imaging in detecting malignant effusion were 75.0%, 91.7% and 93.5%, respectively, which were 69.8%, 91.9% and 93.0% in distinguishing metastatic effusion. The sensitivity of ^18^F-FDG PET/CT integrated imaging in detecting malignant effusion was higher than that of CT imaging (p = 0.000). For metastatic effusion, ^18^F-FDG PET imaging had higher sensitivity (p = 0.000) and better diagnostic consistency with ^18^F-FDG PET/CT integrated imaging compared with CT imaging (Kappa = 0.917 and Kappa = 0.295, respectively). The specificities of CT imaging, ^18^F-FDG PET imaging and ^18^F-FDG PET/CT integrated imaging were 94.1%, 63.2% and 92.6% in detecting benign effusion. The specificities of CT imaging and ^18^F-FDG PET/CT integrated imaging were higher than that of ^18^F-FDG PET imaging (p = 0.000 and p = 0.000, respectively), and CT imaging had better diagnostic consistency with ^18^F-FDG PET/CT integrated imaging compared with ^18^F-FDG PET imaging (Kappa = 0.881 and Kappa = 0.240, respectively).

**Conclusion:**

^18^F-FDG PET/CT integrated imaging is a more reliable modality in distinguishing malignant from benign pleural effusion than ^18^F-FDG PET imaging and CT imaging alone. For image interpretation of ^18^F-FDG PET/CT integrated imaging, the PET and CT portions play a major diagnostic role in identifying metastatic effusion and benign effusion, respectively.

## Introduction

Pleural effusion caused by a number of malignant and benign diseases is a common and challenging medical problem. In clinic, a series of diagnostic assessments are of great importance for the rational treatment. Above all, differential diagnosis that distinguishes malignant from benign pleural effusion is the first problem which has to be solved. Computed tomography (CT) and positron emission tomography (PET) as noninvasive methods have been used in characterizing pleural effusion as malignancy or benignancy, and can trigger the determination of etiology in some cases [1,2]. PET/CT scanner combining in-line PET and CT cameras provides functional and anatomic/morphologic imagings, and is a new device with considerable diagnostic potential [3]. Several studies have been implemented to discuss the role of PET/CT using ^18^F-fluoro-2-deoxy-D-glucose (^18^F-FDG) in assessing the nature of pleural effusion [4,5,6,7,8]. However, their purposes were to evaluate a series of parameters such as density of the effusion, morphology of any solid pleural abnormality and increased uptake of ^18^F-FDG in pleural effusion and pleura, and the solid pleural abnormality and pleural uptake were significant parameters used for differentiation [4,5]. However, their results were displayed in the form of ^18^F-FDG PET or CT alone. So far, the method that integrates pleural abnormality on PET imaging with that on CT imaging in the differential diagnosis of pleural effusion has not been explored. We performed this study to determine whether ^18^F-FDG PET/CT integrated imaging could effectively distinguish malignant from benign pleural effusion. In addition, the diagnostic role of CT and PET portions from ^18^F-FDG PET/CT integrated imaging remains to be inexplicit, when interpreting the diagnostic results of ^18^F-FDG PET/CT integrated imaging. Thus, the other purpose of the current study was to investigate the diagnostic role of CT and PET portions from ^18^F-FDG PET/CT integrated imaging in the differential diagnosis of pleural effusion.

## Materials and Methods

### Patient selection

We conducted a retrospective study of patients with pleural effusion from June 2005 to May 2013, who underwent ^18^F-FDG PET/CT examination to differentiate malignancy from benignancy. Patients enrolled in the present study met the following inclusion criteria. The definitive pathologic diagnosis of malignant pleural effusion was confirmed by thoracocentesis, pleural sample from needle biopsy, thoracoscope or thoracotomy performed within 4 weeks after ^18^F-FDG PET/CT examination. None of enrolled patients with malignant pleural effusion had received induction chemotherapy and/or radiation therapy before ^18^F-FDG PET/CT examination. Benign pleural effusion was concluded by thoracoscope, thoracotomy or clinical findings. On the basis of clinical findings, benign pleural effusion was confirmed if ⑴ at least 2 cytologic results were negative for malignancy, and ⑵ there were clinical findings of a disease such as inflammatory pleural effusion, tuberculous pleural effusion, and transudate caused by congestive heart failure, renal insufficiency, or hepatic cirrhosis, and ⑶ pleural effusion disappeared after symptomatic treatment and clinical follow-up was over 12 months. Finally, a total of 176 patients with pleural effusion (108 men and 68 women; age range, 35–92 years; mean age ± SD, 60 ± 12 years) were enrolled in the study. The present study was approved by Institutional Research Board of Harbin Medical University.

### PET/CT scanning

All patients involved in the study were required to fast for 4~6 hours before subjecting to whole-body ^18^F-FDG PET/CT examination (Discovery ST; General Electric Medical System). Blood glucose level of all patients was lower than 7 mmol/L before receiving the tracer. The average dosage of ^18^F-FDG was from 4 to 5 MBq/Kg of body weight. After ^18^F-FDG injection, the patients were required to keep supine for 60 min in a quiet room without being disturbed. The scanning range was from head to proximal thigh. The CT scanning was performed with 120 kV, 150 mA, 0.8 seconds per CT rotation and 3.75mm slice thickness. PET scanning was performed with the same position in the 2-dimensional mode. The emission scan time was 2.5 min per bed position, and 6–7 bed positions were generally taken for all patients. Attenuation correction for PET was based on the CT data. Transaxial, sagittal, and coronal images were analyzed on computer workstation (Xeleris; General Electric Medical System).

### Interpretation of imaging

The images from all patients with pleural effusion were reviewed by two experienced physicians in PET/CT who were unaware of the results of pathology and follow-up, and the final results of image assessment were based on the consensus reading. On computer workstation, the nature of pleural effusion was assessed on the basis of the pleural abnormality.

Concerning CT image interpretation, the suspected malignant pleural effusion was based on the presence of at least one criterion for malignant pleural thickening reported by Traill *(1)*: nodular or focal pleural thickening, and irregular pleural thickening. In contrast, pleural effusion with no pleural thickening or diffuse smooth thickening was regarded as benign disease.

Images of ^18^F-FDG PET were analyzed by visual interpretation. In brief, the degree of ^18^F-FDG uptake in pleural region was compared with background mediastinal activity. Any pleural lesion with increased ^18^F-FDG uptake higher than mediastinal activity was interpreted as malignant pleural effusion. ^18^F-FDG PET suggested negative result if pleural activity was equal to or less than mediastinal activity. Meanwhile, the biodistribution pattern of positive pleural activity was defined as diffuse, nodular and multiple nodular lesion, and the maximal standardized uptake values (SUVmax) were calculated by overlaying region of interest (ROI) onto positive pleura.

**Fig 1:**
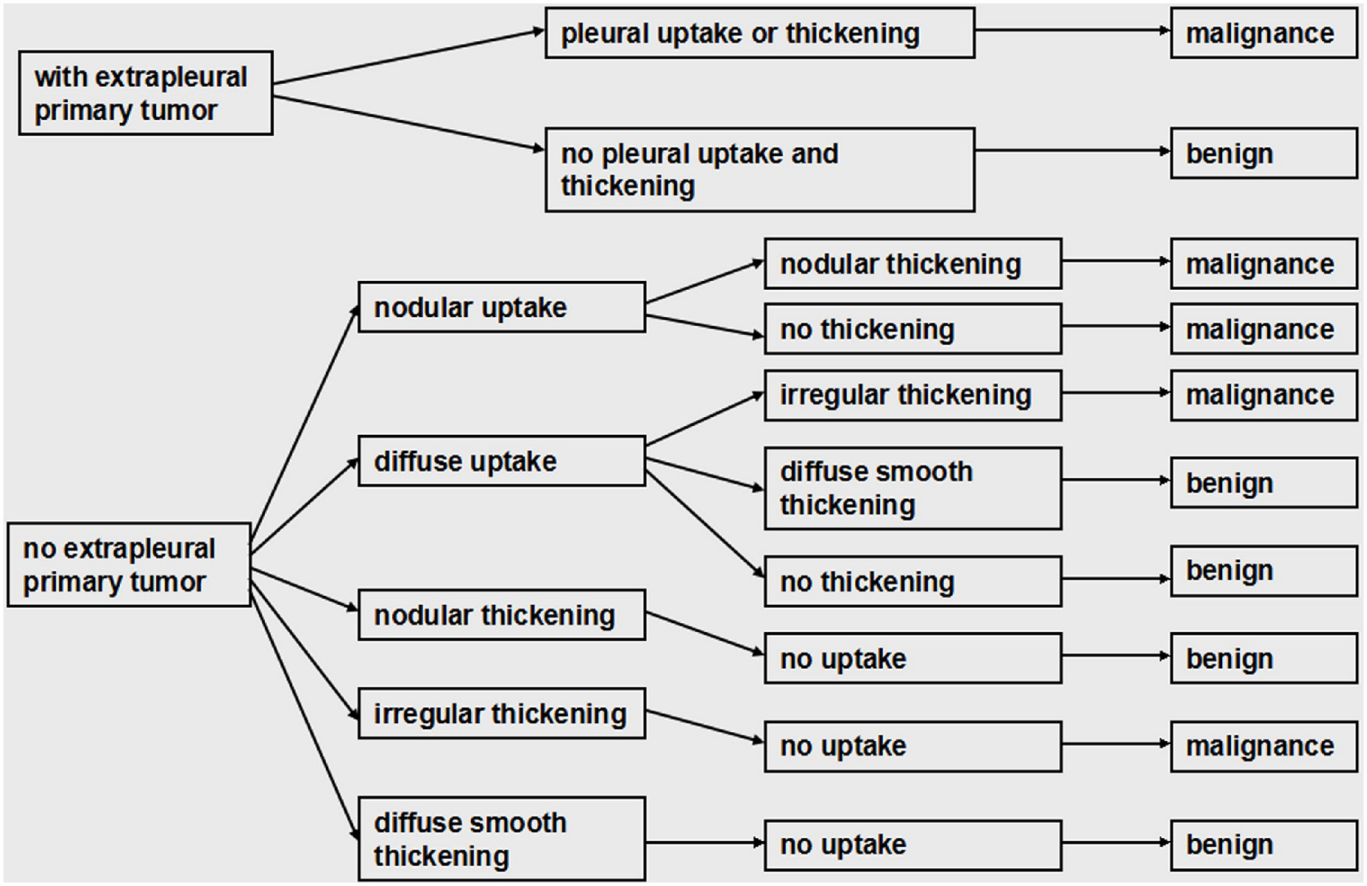
The diagnostic criteria of ^18^F-FDG PET/CT integrated imaging in the differential diagnosis of pleural effusion.

^18^F-FDG PET/CT integrated imaging was interpreted by combining the morphologic feature of the pleura on CT imaging with the degree and form of pleural ^18^F-FDG uptake on PET imaging. The first step was to determine whether there was a suspicious extrapleural primary malignancy. Afterwards, image interpretation was based on the diagnostic criteria showed in Fig 1.

### Statistical analysis

The diagnostic results of CT imaging, ^18^F-FDG PET imaging and ^18^F-FDG PET/CT integrated imaging were compared with pathology and clinical findings, and the sensitivities and specificities of three imagings were evaluated. SPSS 16.0 software package (SPSS Inc., Chicago, Illinois, USA) was used in this study. (1) McNemar test was used to compare the differences between the sensitivity and specificity of ^18^F-FDG PET/CT integrated imaging and those of CT imaging and ^18^F-FDG PET imaging alone to investigate whether ^18^F-FDG PET/CT integrated imaging could more effectively separate pleural effusion into malignancy and benignancy. A *p* value of less than 0.05 was considered to be statistical significant. (2) To survey the diagnostic role of CT and PET portions from ^18^F-FDG PET/CT integrated imaging in the antidiastole of pleural effusion, the sensitivity and specificity of ^18^F-FDG PET imaging were compared with those of CT imaging alone using McNemar test, and Measure of Agreement Kappa was applied to test the diagnostic consistency between CT imaging and ^18^F-FDG PET/CT integrated imaging, and that between ^18^F-FDG PET imaging and ^18^F-FDG PET/CT integrated imaging. The weighted Kappa value for the degree of agreement was acquired, and higher Kappa value represented better consistency, suggesting better diagnostic role.

## Results

### Pathologic and clinical findings

Among 176 patients, 108 were found to have malignant pleural effusion, which were confirmed by thoracentesis (n = 50), needle biopsy (n = 48), direct thoracoscopic biopsy (n = 7) and exploratory thoracotomy (n = 3). Primary tumors were as follows: pleural mesothelioma (n = 22), lung cancer (n = 77), ovarian cancer (n = 4), pancreatic cancer (n = 3), renal carcinoma (n = 1), and hepatocarcinoma (n = 1). The other 68 patients had benign pleural effusion, including tuberculous pleural effusion (n = 30), inflammatory effusion (n = 26), hepatic cirrhosis (n = 2), cardiogenic effusion (n = 8), and renal origin (n = 2), which were confirmed by thoracoscope (n = 5), thoracotomy (n = 4), needle biopsy (n = 3) or clinical examinations (n = 56).

### Diagnostic role of CT imaging, PET imaging and PET/CT integrated imaging in evaluating malignant pleural effusion

Eighty one patients were diagnosed as malignant pleural effusion on CT imaging, including 21 cases of pleural mesothelioma and 60 cases of pleural metastasis. However, the other 27 patients with malignant pleural effusion caused by 1 case of pleural mesothelioma and 26 cases of pleural metastasis were misdiagnosed as benignancy. The sensitivity of CT imaging in detecting malignant pleural effusion was 75.0% (81/108), and the sensitivities of CT imaging in detecting malignant pleural effusion caused by pleural mesothelioma and pleural metastasis were 95.5% (21/22) and 69.8% (60/86), respectively.

Based on ^18^F-FDG PET findings, 99 patients with malignant effusion caused by 20 cases of pleural mesothelioma and 79 cases of pleural metastasis were correctly identified in terms of increased pleural ^18^F-FDG uptake with SUVmax of 5.9±2.8 (range 2.2 to 15.3). The biodistribution pattern of ^18^F-FDG included 80 cases of diffuse uptake, 8 cases of nodular distribution and 11 cases of multiple nodular uptake. While 9 patients with malignant effusion (2 had pleural mesothelioma and 7 had pleural metastasis) were read to be negative due to low pleural uptake. The sensitivity of ^18^F-FDG PET imaging in detecting malignant pleural effusion was 91.7% (99/108), and the sensitivities of ^18^F-FDG PET imaging in detecting pleural effusion caused by pleural mesothelioma and pleural metastasis were 90.9% (20/22) and 91.9% (79/86), respectively.

**Fig 2:**
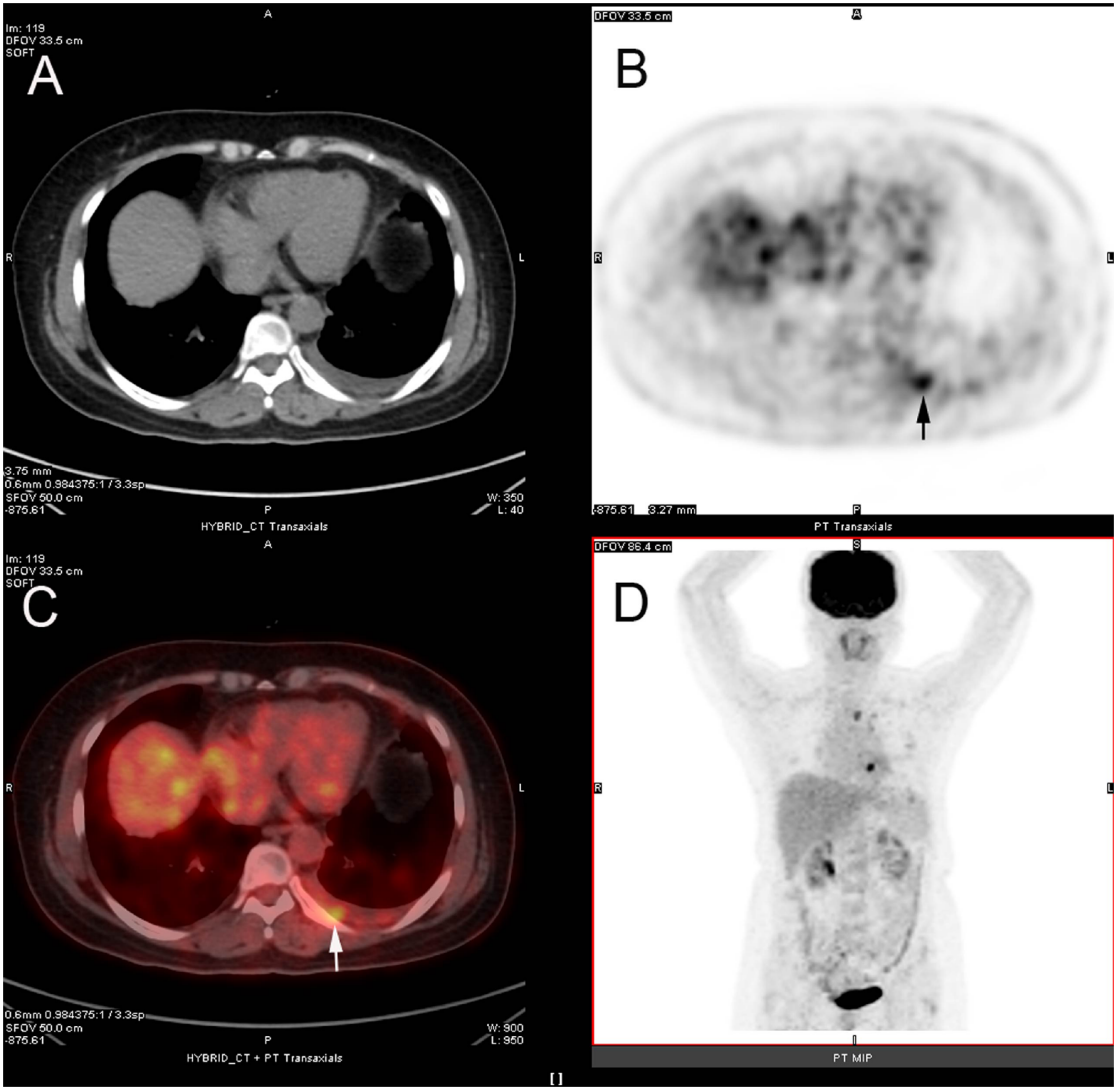
^18^F-FDG PET/CT integrated imaging of 54-year old woman with left lung cancer and malignant pleural effusion. Axial CT (A) shows effusion in left pleural cavity, and axial ^18^F-FDG PET (B, arrow) and axial fused ^18^F-FDG PET/CT (C, arrow) display nodular ^18^F-FDG uptake (SUVmax of 3.0) in left-posterior pleural region. Pathology from thoracentesis confirmed malignant pleural effusion caused by metastatic adenocarcinoma.

^18^F-FDG PET/CT integrated imaging correctly detected the presence of malignant pleural effusion in 101 patients, including 21 cases of pleural mesothelioma and 80 cases of pleural metastasis. In these patients, PET and CT imagings simultaneously supported malignant pleural effusion in 20 cases of pleural mesothelioma and 59 cases of pleural metastasis. However, CT imaging displayed malignant pleural effusion in 1 case of pleural mesothelioma and 1 case of pleural metastasis with negative findings on PET imaging, while 20 patients with malignant pleural effusion caused by pleural metastasis were diagnosed as malignancy on PET imaging with negative findings on CT imaging (Fig 2). The remaining patients with malignant pleural effusion (1 pleural mesothelioma and 6 pleural metastasis) were falsely evaluated on ^18^F-FDG PET/CT integrated imaging. The sensitivity of ^18^F-FDG PET/CT integrated imaging in detecting malignant pleural effusion was 93.5% (101/108), and the sensitivities of ^18^F-FDG PET/CT integrated imaging in detecting pleural effusion caused by pleural mesothelioma and pleural metastasis were 95.5% (21/22) and 93.0% (80/86), respectively.

As shown in Table 1, the sensitivities of both ^18^F-FDG PET imaging and ^18^F-FDG PET/CT integrated imaging in detecting malignant pleural effusion were higher than that of CT imaging alone (p = 0.000 and p = 0.000, respectively). Further classification analysis displayed that there was no statistical difference among the sensitivities of CT, ^18^F-FDG PET and ^18^F-FDG PET/CT integrated imaging in distinguishing malignant effusion caused by pleural mesothelioma (p = 1.000, p = 1.000 and p = 1.000, respectively), and the diagnostic consistency between CT imaging and ^18^F-FDG PET/CT integrated imaging was better than that between ^18^F-FDG PET imaging and ^18^F-FDG PET/CT integrated imaging (Kappa = 1.000 and Kappa = 0.645, respectively). While for metastatic pleural effusion, the sensitivities of ^18^F-FDG PET imaging and ^18^F-FDG PET/CT integrated imaging were higher than that of CT imaging (p = 0.000 and p = 0.000, respectively), and the diagnostic consistency between ^18^F-FDG PET imaging and ^18^F-FDG PET/CT integrated imaging was obviously superior to that between CT imaging and ^18^F-FDG PET/CT integrated imaging (Kappa = 0.917 and Kappa = 0.295, respectively).

**Table 1:** The efficacy of CT, PET and PET/CT integrated imaging in antidiastole of pleural effusion.

Modality	Sensitivity			Specificity
	Total	Pleural mesothelioma	Pleural metastasis	
**CT**	**75.0℅**	**95.5℅**	**69.8℅**	**94.1℅**
**PET**	**91.7℅**	**90.9℅**	**91.9℅**	**63.2℅**
**PET/CT**	**93.5℅**	**95.5℅**	**93.0℅**	**92.6℅**
p(CT*PET/CT)	**0.000**	**1.000**	**0.000**	**1.000**
p(PET*PET/CT)	**0.500**	**1.000**	**1.000**	**0.000**
p(CT*PET)	**0.000**	**1.000**	**0.000**	**0.000**
Kappa(CT*PET/CT)	**0.344**	**1.000**	**0.295**	**0.881**
Kappa(PET*PET/CT)	**0.865**	**0.645**	**0.917**	**0.240**

p(CT*PET/CT), results of McNemar test between CT and PET/CT; p(PET*PET/CT), results of McNemar test between PET and PET/CT; p(CT*PET), results of McNemar test between CT and PET; Kappa(CT*PET/CT), results of Measure of Agreement Kappa between CT and PET/CT; Kappa(PET*PET/CT), results of Measure of Agreement Kappa between PET and PET/CT.

### Diagnostic role of CT imaging, PET imaging and PET/CT integrated imaging in distinguishing benign pleural effusion

Of 68 patients with benign pleural effusion, 64 were correctly identified according to CT imaging alone, which resulted in a specificity of 94.1%. ^18^F-FDG PET imaging correctly characterized 43 patients for the absence of ^18^F-FDG uptake within the pleura, while 8 cases of inflammatory effusion and 17 cases of tuberculous pleural effusion were mistakenly considered to be malignant due to high pleural ^18^F-FDG uptake. SUVmax of positive pleura was 5.4±2.0 (range 2.2 to 11.8). This resulted in a specificity of 63.2% (43/68) on ^18^F-FDG PET imaging in distinguishing benign pleural effusion.

**Fig 3:**
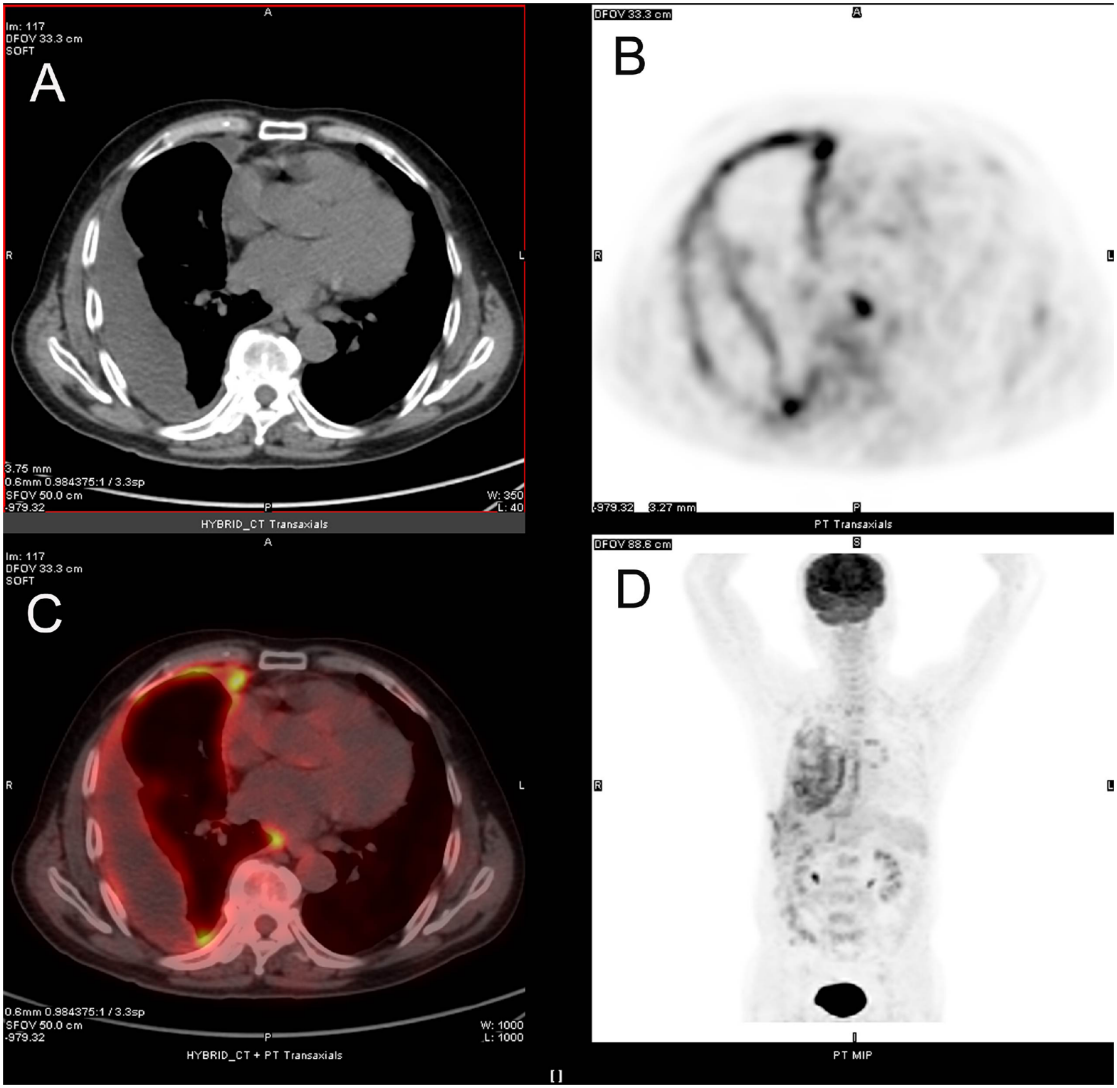
^18^F-FDG PET/CT integrated imaging of 72-year old man with tuberculous pleural effusion. Axial CT (A) shows effusion in right pleural cavity and diffuse light smooth thickening of the pleura. Axial ^18^F-FDG PET (B) and axial fused ^18^F-FDG PET/CT (C) display diffuse ^18^F-FDG uptake in right pleural region (SUVmax of 7.9).

^18^F-FDG PET/CT integrated imaging correctly assessed benign pleural effusion in 63 patients. Among these patients, 43 were simultaneously regarded as benignancy on both CT and PET imagings, while 8 cases of inflammatory effusion and 12 cases of tuberculous pleural effusion showing false-positive findings on ^18^F-FDG PET imaging were interpreted to be benign by means of combining morphologic manifestation of CT imaging with no sign of malignancy (Fig 3). Four patients with tuberculous pleural effusion were regarded as malignancy in view of malignant signs on both CT and PET imagings. The remaining 1 patient with tuberculous pleural effusion was interpreted as malignancy due to multiple nodular ^18^F-FDG uptake of pleura with negative finding on CT imaging. The specificity of ^18^F-FDG PET/CT integrated imaging in detecting benign pleural effusion was 92.6% (63/68).

There was no pleural thickening on CT imaging and no pleural ^18^F-FDG uptake on PET imaging in 12 patients with pleural effusion caused by the dysfunction of liver, heart and kidney. Of 26 patients with inflammatory effusion, 7 had encapsulated effusion and 19 had free pleural effusion. Five out of these patients with encapsulated effusion showed diffuse ^18^F-FDG retention in the pleura encompassing effusion, and 3 of 19 patients with free pleural effusion had diffuse ^18^F-FDG uptake of the pleura on PET imaging. In these patients with pleural ^18^F-FDG uptake, light smooth thickening of the pleura was displayed on the corresponding region of CT imaging.

For 30 patients with tuberculous pleural effusion, 3 showed encapsulated effusion and 27 displayed free effusion. Pleural ^18^F-FDG retention on PET imaging was present in 17 patients, including 13 with diffuse ^18^F-FDG uptake, 2 with diffuse ^18^F-FDG uptake at costodiaphragmatic recesses, 1 with nodular ^18^F-FDG uptake in pleural reflection and 1 with multiple nodular ^18^F-FDG uptake. The detailed image features of CT and PET imagings of tuberculous pleural effusions are showed in Table 2.

**Table 2:** Summary of pleural features on CT and PET imagings of 30 tuberculous pleural effusions.

Characteristic	No. of patients(n)	PET findings	CT findings	Interpretation results of PET/CT
**Encapsulated effusion**	**1**	**no uptake**	**slight smooth thickening of encapsulated pleura**	**benign**
	**2**	**diffuse uptake in encapsulated pleura**	**slight smooth thickening of encapsulated pleura**	**benign**
**Free effusion**	**1**	**nodular uptake**	**nodular thickening**	**malignant**
	**3**	**diffuse uptake**	**irregular thickening**	**malignant**
	**1**	**multiple nodular uptake**	**no thickening**	**malignant**
	**2**	**diffuse uptake at costodiaphragmatic recesses**	**no thickening or slight smooth thickening**	**benign**
	**8**	**diffuse uptake**	**slight diffuse smooth thickening**	**benign**
	**5**	**no pleural uptake**	**slight diffuse smooth thickening**	**benign**
	**7**	**no pleural uptake**	**no thickening**	**benign**

The specificities of CT imaging and ^18^F-FDG PET/CT integrated imaging were higher than that of ^18^F-FDG PET imaging (p = 0.000 and p = 0.000, respectively). There was no statistical difference of specificity between CT imaging and ^18^F-FDG PET/CT integrated imaging (p = 1.000). The diagnostic consistency between CT imaging and ^18^F-FDG PET/CT integrated imaging was superior to that between ^18^F-FDG PET imaging and ^18^F-FDG PET/CT integrated imaging (Kappa = 0.881 and Kappa = 0.240, respectively) (Table 1).

## Discussion

^18^F-FDG PET/CT imaging as a method of differential diagnosis has been reported to distinguish malignant from benign pleural effusion [4,5,6,7], and the presences of pleural abnormality on CT imaging and pleural region uptake on ^18^F-FDG PET imaging are found to be the most accurate criteria in determining the malignant nature of pleural effusion [4,5]. However, the efficacy of ^18^F-FDG PET/CT imaging are displayed in the form of ^18^F-FDG PET and CT alone. For instance, Toaff et al. [4] reported a ^18^F-FDG PET/CT study of 31 patients with primary extrapleural malignancy and pleural effusion, and found that the sensitivities of pleural uptake on PET imaging and pleural lesion on CT imaging were 86% and 71%, respectively, and the specificity was 90% for both of the 2 parameters. Kim et al. [5] reported that the sensitivity and specificity of ^18^F-FDG PET imaging was 87.5% and 88.8%, respectively, and the respective sensitivity and specificity of CT imaging was 83.3% and 88.8%. The difference of the present study from previous studies was the method for image interpretation of ^18^F-FDG PET/CT imaging in identifying the nature of pleural effusion, and the diagnosis results of ^18^F-FDG PET/CT imaging were determined by synthesizing the degree and form of pleural ^18^F-FDG uptake on PET imaging and pleural morphologic manifestation on CT imaging. By this means, we found that ^18^F-FDG PET/CT integrated imaging with sensitivity of 93.5% and specificity of 92.6% showed superior sensitivity to CT imaging alone and higher specificity than PET imaging alone, respectively. The work presented here demonstrated that ^18^F-FDG PET/CT integrated imaging was a more reliable diagnostic method than ^18^F-FDG PET and CT imagings in the differential diagnosis of pleural effusion.

The present study also investigated the diagnostic role of CT and PET portions from ^18^F-FDG PET/CT integrated imaging in the antidiastole of pleural effusion. In our study, malignant effusion was divided into the groups of pleural mesothelioma and metastatic pleurisy in the light of primary cause. In detecting malignant effusion caused by pleural mesothelioma, CT imaging and ^18^F-FDG PET imaging had relatively high sensitivity. Moreover, CT imaging had better diagnostic consistency with ^18^F-FDG PET/CT integrated imaging than PET imaging. These findings suggest that CT portion from ^18^F-FDG PET/CT integrated imaging played a major role in distinguishing malignant effusion caused by pleural mesothelioma.

In contrast to pleural effusion caused by mesothelioma, metastatic pleural effusion has higher morbidity. CT imaging can detect subtle pleural metastases without or with a small amount of associated pleural effusion [9], and thin-section CT imaging provides more useful information than thick-section CT imaging for the evaluation of pleural dissemination with sensitivity of 90% [10]. However, in this study, the morphologic imaging on unenhanced CT imaging alone could not effectively distinguish metastatic pleural effusion with sensitivity of 69.8%. This implies that pleural effusion is the dominant factor that affects CT imaging to detect pleural metastases. ^18^F-FDG PET imaging, revealing metabolic activity of disease rather than its morphologic structure, is not affectted by pleural effusion and can play a significant role in lung cancer patients with pleural effusion and normal or equivocal involvement of pleural surface on CT imaging [2,11]. A previous study showed that the sensitivity of ^18^F-FDG PET imaging in identifying pleural involvement in patients with pleural effusion and extrapleural malignancy, including lung cancer, lymphoma, melanoma and ovarian cancer, was greater than that of CT imaging alone [4]. This was consistent with our results, although the types of primary tumor in this study were different from previous researches. Meanwhile, ^18^F-FDG PET imaging had higher diagnostic consistency with ^18^F-FDG PET/CT integrated imaging compared with CT imaging. Therefore, when ^18^F-FDG PET/CT integrated imaging was used to indentify the nature of pleural effusion, we mainly taked advantage of the ability of ^18^F-FDG PET portion to detect pleural metastases.

^18^F-FDG PET imaging has been reported to possess limited specificity in differentiating malignant from benign pleural effusion [6]. Low specificity of ^18^F-FDG PET imaging associated with the pleural false-positive findings caused by benign diseases, similar to changes seen on malignancy, is never effectively resolved, which results in more unnecessary invasive examinations such as thoracoscope, thoracocentesis and needle biopsy of pleura. Some studies have previously focused on the diagnostic effect of PET imaging and the exact anatomic location of CT imaging, when interpreting diagnosis results of ^18^F-FDG PET/CT integrated imaging [8,12]. However, based on our experience, the diagnostic role of CT imaging to the management of pleural effusion should’t be neglected, and the CT portion from ^18^F-FDG PET/CT integrated imaging played a major diagnostic role in identifying benign pleural effusion. The results of our study showed that CT imaging had higher specificity and better diagnostic consistency with ^18^F-FDG PET/CT integrated imaging compared with ^18^F-FDG PET imaging. In interpreting images of ^18^F-FDG PET/CT integrated imaging, CT imaging could be used to correct the false-positive findings on PET imaging and enhance the diagnosis specificity with the sensitivity not being reduced. Eight cases of inflammatory effusion and 12 cases of tuberculous pleural effusion with false-positive findings on ^18^F-FDG PET imaging were finally diagnosed as benign disorder because the malignant diagnosis was not accepted on CT imaging.

For inflammatory effusion, there was a tendency that increased pleural ^18^F-FDG uptake was more easily found in encapsulated pleural effusion (5/7) compared with free pleural effusion (3/19). This may be ascribed to more serious inflammatory reaction of the pleura encompassing encapsulated effusion. However, the pleura with false-positive findings on PET imaging displayed diffuse ^18^F-FDG uptake accompany with light smooth thickening of the corresponding region on CT imaging.

It is common that false positivity on PET imaging is caused by tuberculous disease [13,14,15]. Orki et al. [8] reported that patients with tuberculous pleuritis could have region of increased ^18^F-FDG uptake in the pleura. In this study, 56.7% (17/30) of tuberculous pleural effusion showed false-positive findings on PET imaging, most of which displayed diffuse ^18^F-FDG uptake in the pleural region.

Several limitations probably influenced this study. Firstly, in the study, unenhanced CT was used to evaluate the nature of pleural effusion. However, contrast-enhanced CT is superior to unenhanced CT in differentiating pleural thickening from effusion. So, this may affect the role of CT in identifying malignant pleural effusion. Secondly, the differential diagnosis of pleural effusion in the present study was performed on the basis of the pleural abnormality. However, the ^18^F-FDG uptake degree of effusion may be useful in evaluating the nature of pleural effusion. Because cast-off cells of tumor in effusion also cause ^18^F-FDG uptake, which may be different from benign pleural effusion.

## Conclusion

In differentiating malignant from benign pleural effusion, ^18^F-FDG PET/CT integrated imaging is a more useful modality than ^18^F-FDG PET imaging and CT imaging alone. When interpreting images of ^18^F-FDG PET/CT integrated imaging, the PET and CT portions play a major role in detecting metastatic effusion and benign effusion, respectively. CT imaging can be used to correct the false-positive findings on PET imaging and improve the specificity with the sensitivity not being reduced.

## Supporting Information

S1 File Original data of the present study. (XLS)

S2 File The letter of approval from Institutional Research Board of Harbin Medical University. (DOC)
