# Supplementary figures and images for: The Role of 18F-FDG PET/CT Integrated Imaging in Distinguishing Malignant from Benign Pleural Effusion
Source: PLoS One. 2016 Aug 25;11(8):e0161764. doi: 10.1371/journal.pone.0161764 (PMC4999143; doi:10.1371/journal.pone.0161764)

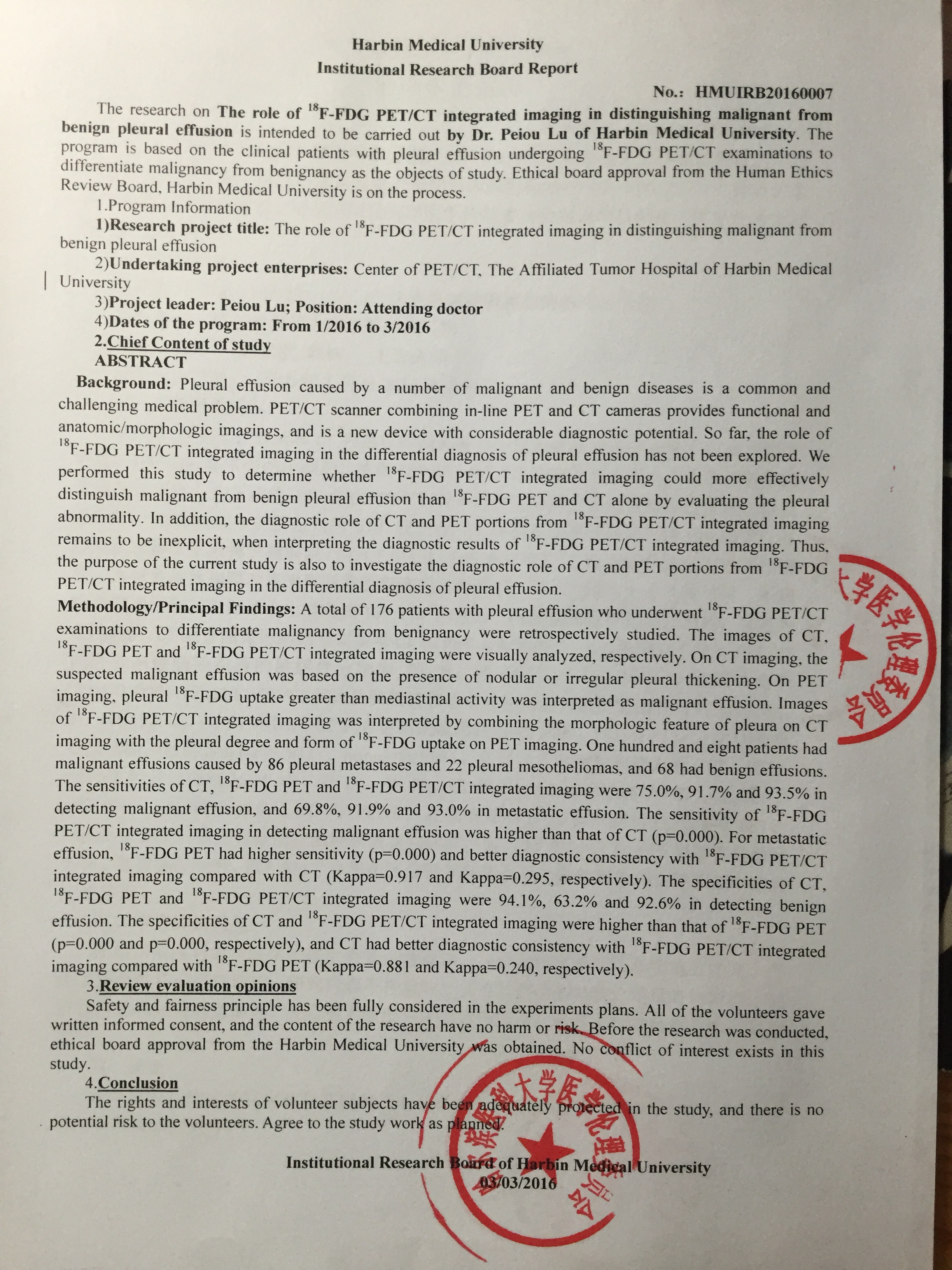


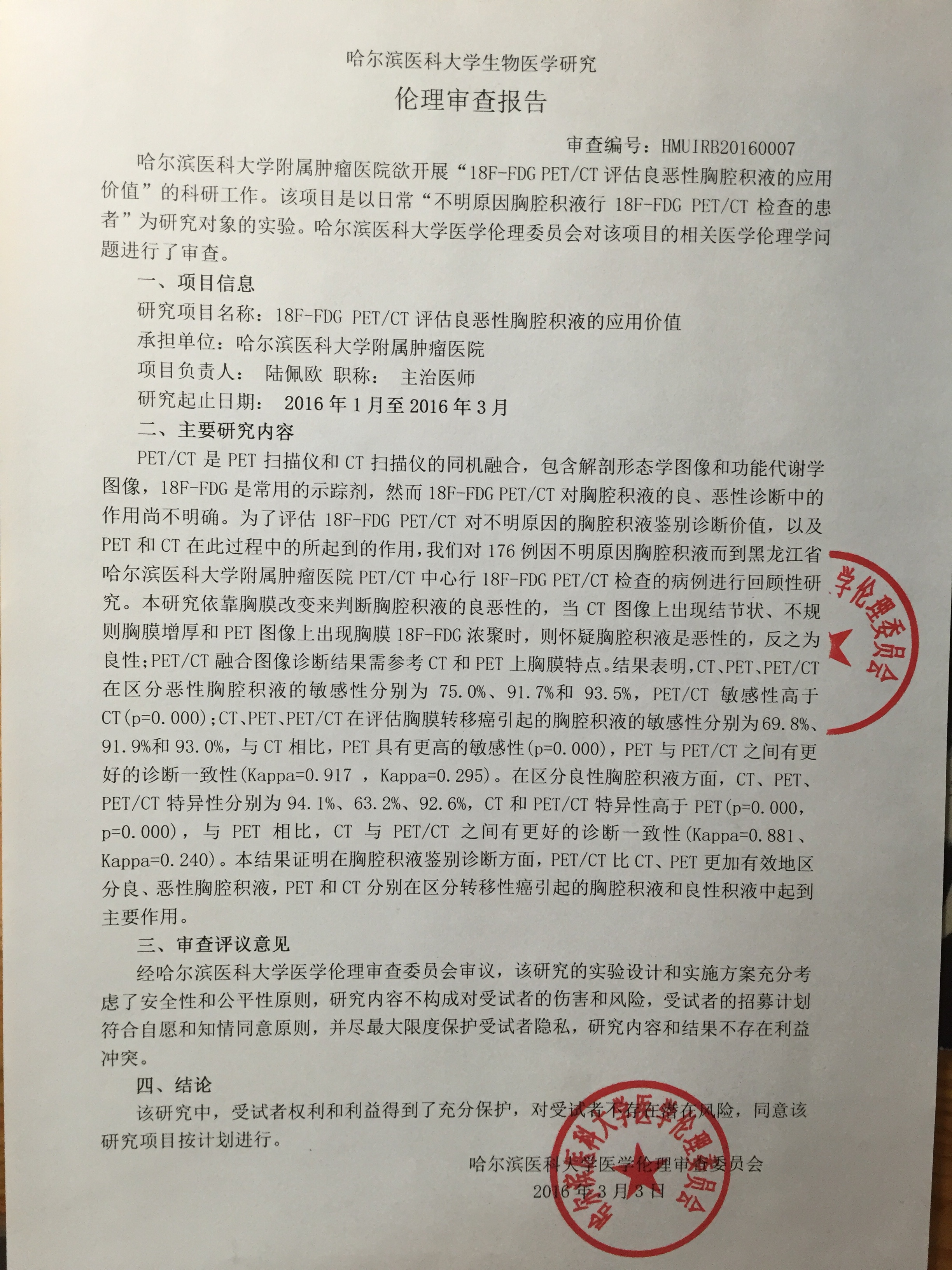

Supplement: S2 File — (DOC) [file pone.0161764.s002.doc]
